# Re-Differentiation Capacity of Human Chondrocytes in Vitro Following Electrical Stimulation with Capacitively Coupled Fields

**DOI:** 10.3390/jcm8111771

**Published:** 2019-10-24

**Authors:** Simone Krueger, Sophie Achilles, Julius Zimmermann, Thomas Tischer, Rainer Bader, Anika Jonitz-Heincke

**Affiliations:** 1 Department of Orthopedics, Rostock University Medical Centre, 18057 Rostock, Germany; sophie.achilles@uni-rostock.de (S.A.); thomas.tischer@gmx.net (T.T.); rainer.bader@med.uni-rostock.de (R.B.); anika.jonitz-heincke@med.uni-rostock.de (A.J.-H.); 2 Institute of General Electrical Engineering, University of Rostock, 18059 Rostock, Germany; julius.zimmermann@uni-rostock.de

**Keywords:** cartilage lesion, electrical stimulation, capacitively coupled electric field, regenerative medicine, chondrocytes

## Abstract

Treatment of cartilage lesions remains a clinical challenge. Therefore, biophysical stimuli like electric fields seem to be a promising tool for chondrocytic differentiation and treatment of cartilage lesions. In this in vitro study, we evaluated the effects of low intensity capacitively coupled electric fields with an alternating voltage of 100 mV_RMS_ (corresponds to 5.2 × 10^−5^ mV/cm) or 1 V_RMS_ (corresponds to 5.2 × 10^−4^ mV/cm) with 1 kHz, on human chondrocytes derived from osteoarthritic (OA) and non-degenerative hyaline cartilage. A reduction of metabolic activity after electrical stimulation was more pronounced in non-degenerative cells. In contrast, DNA contents in OA cells were significantly decreased after electrical stimulation. A difference between 100 mV_RMS_ and 1 V_RMS_ was not detected. However, a voltage-dependent influence on gene and protein expression was observed. Both cell types showed increased synthesis rates of collagen (Col) II, glycosaminoglycans (GAG), and Col I protein following stimulation with 100 mV_RMS_, whereas this increase was clearly higher in OA cells. Our results demonstrated the sensitization of chondrocytes by alternating electric fields, especially at 100 mV_RMS_, which has an impact on chondrocytic differentiation capacity. However, analysis of further electrical stimulation parameters should be done to induce optimal hyaline characteristics of ex vivo expanded human chondrocytes.

## 1. Introduction

Hyaline cartilage is of high importance for the functionality of joints, it ensures a smooth and lubricated friction surface as well as the transfer of loads to the subchondral bone [1,2,3]. For these properties, the viscoelastic characteristic of cartilage, which is mainly mediated by the extracellular matrix (ECM), is essential. Further characteristics of hyaline cartilage are the limited potential for self-repair and that nutrient and oxygen supply is ensured only through fluid movement by diffusion and mechanical loading [1,4].

In contrast to bone, mature hyaline cartilage cannot regenerate itself after traumatic lesions or tissue degeneration due to various reasons like lack of vascular supply and low cell turnover [5]. For this reason, the cartilage tissue is often irreversibly damaged by trauma and by subsequent degenerative processes [6]. Multiple surgical techniques for the treatment of cartilage defects are used depending mainly on the defect size: osteochondral autograft and allograft transplantation, microfracturing, matrix augmented bone marrow stimulation and autologous chondrocyte implantation (ACI) as well as matrix-assisted ACI (MACI). For ACI, autologous chondrocytes are isolated from hyaline cartilage tissue derived from non-weight-bearing joint regions and expanded during ex vivo cultivation. After expansion, the cartilaginous cells are re-implanted in damaged regions of the articular cartilage. In MACI treatment, the expanded chondrocytes are seeded onto a three-dimensional (3D) scaffold before re-implantation to provide a more physiological environment, thus resulting in improved cell integration in the defect and regeneration of cartilage tissue [7].

However, the main problem of ex vivo expansion and cultivation remains the de-differentiation of chondrocytes in monolayer cell cultures, which makes them lose their chondrogenic phenotype [8,9,10]. Using these de-differentiated cells, reconstruction of cartilage lesions is often accompanied by the formation of fibrocartilage tissue [11]. This tissue, in turn, has a lower ability to withstand mechanical stresses during joint loading [12]. Therefore, the treatment of cartilage lesions still remains a major clinical challenge. The current therapy options lack initiation of a healing process or ensurance of the necessary re-differentiation of de-differentiated chondrocytes and therefore do not offer an optimum quality of regenerated tissue [7,13].

In order to develop new and improved treatment options, a large field of research has been established. Among others, several scaffold materials for cartilage repair were introduced [14] and the use of mesenchymal stem cells were investigated [15]. Additionally, biophysical stimulation has been suggested for the improvement of tissue engineering approaches for repairing cartilage lesions [16,17,18,19]. Recently, effects of electrical stimulation on cartilage tissue and chondrocytes have been studied and an increased proliferation and matrix synthesis, as well as a reduced matrix degradation, was revealed [17,20]. These effects can probably be attributed to the unique structure as well as biomechanical and electromechanical properties of hyaline cartilage [16,21]. Therefore, electrical stimulation appeared to be a useful biophysical approach to support the re-differentiation of de-differentiated chondrocytes toward the chondrogenic phenotype, thus counteracting the degeneration of hyaline cartilage [17].

In the literature, different approaches have been described for applying electric fields to cells in vitro. These approaches can be divided into direct stimulation, inductive coupling, capacitive coupling, and semi-capacitive coupling. Of these, direct and capacitive applications are mainly used in vitro [22]. To apply direct current, electrodes are placed directly in the cell culture medium, which serves as conductors. The disadvantage of direct coupling is the interaction of the electrode material with the cell culture environment and the possible resulting electrochemical reactions such as Ph change, formation of hydrogen peroxide or reactive oxygen species, which can damage exposed cells. At capacitive coupling opposing electrodes were placed without contact to the conductive medium. The capacitive coupling of the cell culture medium then takes place via the frequency adaptation of the generator in order to generate an electric field. The separation of charges leads to the creation of an electric field between the capacitor plates or electrodes [22]. Since direct currents do not flow through these systems and the electrode, the materials do not react directly with the surrounding environment. Hence, the use of capacitive systems offers advantages with regard to future applications in ex vivo cultivation systems. In previous studies of our working group, we used direct coupling to stimulate bone and cartilage cells resulting in enhanced regenerative capacity [19,23,24,25]. Although direct coupling of electric fields is used for bone regeneration in situ, this approach is not feasible for cartilage tissue formation ex vivo. Therefore, the aim of this study was to establish an experimental setup for testing capacitively coupled alternating electric fields in order to characterize the influence of capacitively coupled alternating electric fields on the re-differentiation of human chondrocytes in vitro.

## 2. Materials and Methods

### 2.1. Test Setup for Capacitively Coupled Electric Field Stimulation

**Figure 1:**
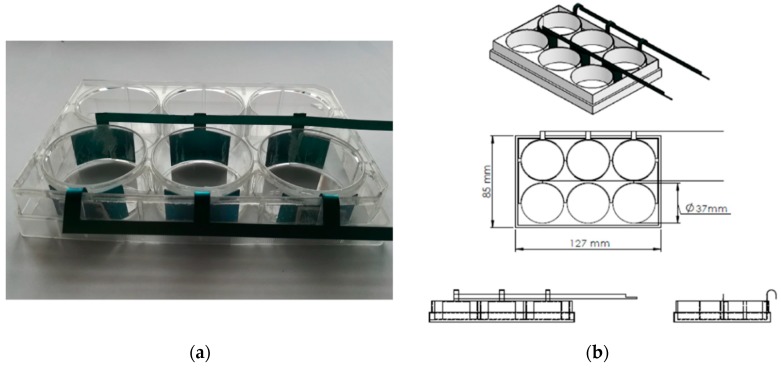
Experimental test setup. (**a**) The processed 6 well tissue culture plate with titanium electrodes. (**b**) Technical drawing of the system for capacitively coupled electric field stimulation.

The experimental system is based on a commercially available six well cell culture plate (Corning Inc., New York, USA). To generate capacitively coupled electric fields, two titanium electrodes (anodized with DOTIZE^®^ by DOT GmbH, Rostock, Germany) were placed opposite to each other on the outside of the well (Figure 1). The three electrodes on each side were connected by the same conductive material as the electrodes. The electrodes were fitted as closely as possible to the polystyrene wells. A notch at the end of the electrodes ensured power supply via attaching a cable with a crocodile clip. A petri dish with fixed cell seeded scaffolds was placed in this system (explained in more detail in Section 2.3).

To apply different sinusoidal alternating voltages with a frequency of 1 kHz, a function generator (GX 310, Metrix, Annecy-le-Vieux, France) was used. A custom-made timer allowed automated stimulation for 45 min three times per day following the protocol of Hiemer et al. [19]. The stimulation system itself was placed in an incubator to provide a stable and hypoxic environment (37 °C, 5% CO_2_ and 5% O_2_) for the cells.

### 2.2. Numerical Simulation of the Capacitively Coupled Electric Field

The finite element simulation package COMSOL Multiphysics^®^, v5.3a (COMSOL AB, Stockholm, Sweden) was used to build a geometric model of the electric fields corresponding to a single well with a pair of electrodes. The double wall resulting from the insertion of the petri dish into the cell culture plate is considered in the simulation. The electric field was computed using the “electric currents” interface at a frequency of 1 kHz. The solution was obtained for a total number of degrees of freedom of 2,713,555. One electrode was set to a potential of 0 V and the other to 1 V, corresponding to a voltage difference of 1 V. Since the system to be solved is linear, the solution and its derived quantities can be computed for any voltage difference by simply multiplying the solution with the ratio of the voltage difference and 1 V. Note that the voltage that is used in COMSOL refers to the amplitude of the signal, whereas lab equipment such as multimeter or function generator often use the RMS voltage. This must be accounted for when calculating the electric field. For example, 100 mV_RMS_ represents an amplitude of 141.4 mV.

**Figure 2:**
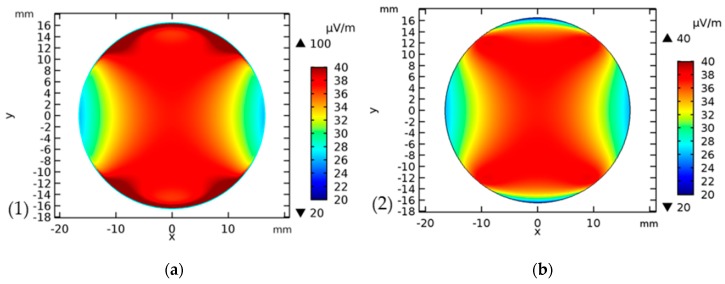
Numerical simulation of electric fields within the experimental test setup. (**a**) The electric field distribution simulated with COMSOL Multiphysics^®^, v5.3a at the bottom of the Petri dish and (**b**) at 1 mm above the bottom for a potential difference of 1 V. At an amplitude of 141.4 mV, the result has to be scaled by a factor of 0.1414, yielding e.g., a maximum field of about 5.7 µV/m instead of 40 µV/m at 1 V amplitude.

The solution for the electric field at the bottom of the cell culture well and the electric field 1 mm above the bottom (corresponding to the location of the cells) is shown in Figure 2. Applying a voltage of 100 mV_RMS_ and a frequency of 1 kHz to our test system resulted in field strength of about 5.2 × 10^−5^ mV/cm, which acted on the cells. Using a voltage 10 times higher, the resulting amplitude on cells was also 10 times higher (5.2 × 10^−4^ mV/cm). The voltage inside the medium was almost constantly equal to 0.5 V, which potentially paved the way for validating the simulation by measuring this voltage.

### 2.3. Cell Culture and Stimulation

Human chondrocytes were isolated either from osteoarthritic or non-degenerative hyaline cartilage. The tissue originates from articular cartilage of the knee joint, which was donated by patients undergoing primary total knee replacement (*n* = 6, 3 female donors: 67 ± 12 years, 3 male donors: 70 ± 7 years) or was post-mortally derived within 72 h after the death of the donors (*n* = 4, 1 female donor 29 years, 3 male donors 43 ± 11 years). The study was approved by the local Ethical Committee of the University of Rostock (registration numbers: A 2009–0017 and A 2011–0138).

Chondrocytes were isolated as described previously [26]. Cells were expanded at 37 °C, 5% CO_2_, and 21% O_2_ and cryopreserved at passage 2. After thawing, the chondrocytes were cultivated in Dulbecco’s Modified Eagle Medium (DMEM, Gibco^®^, Thermo Fisher Scientific Inc., Waltham, MA, USA) with 10% fetal bovine serum (Pan Biotech, Aidenbach, Germany), 1% penicillin/streptomycin (Thermo Scientific, Waltham, MA, USA), 1% Amphotericin B (Biochrom GmbH, Berlin, Germany) and 50 µg/mL ascorbic acid (Sigma-Aldrich, Merck KGaA, Darmstadt, Germany) in a 75 cm^2^ cell culture flask at 37 °C, 5% CO_2_, and 5% O_2_ to reach a confluency of nearly 90%.

At passage three, cells were seeded on a three-dimensional collagen elastin scaffold (Matriderm, MedSkin Solutions Dr. Suwelack AG, Billerbeck, Germany), mainly consisting of bovine collagen type I. The collagen scaffold was punched into round scaffolds with 16 mm in diameter. Using biocompatible silicone adhesive (Korasilon paste, Kurt Obermeier GmbH & Co. KG, Bad Berleburg, Germany), two scaffolds were fixed on the ground of one petri dish (35 mm diameter) (Thermo Scientific, Waltham, MA, USA).

**Figure 3:**
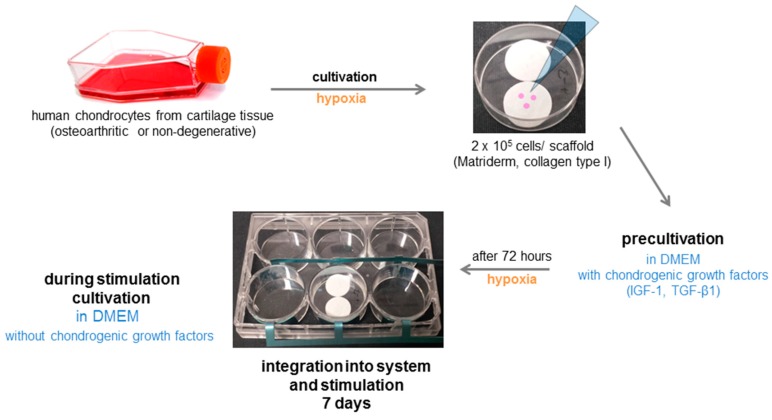
Scheme of the experimental procedure.

On each scaffold, 200,000 cells in 250 µL of medium were seeded. After an initial adherence time of 30 min, each 6-well was filled up with 3 mL DMEM containing 1% Pen/Strep, 1% Amphotericin B, 1% Insulin-Transferrin-Selen (ITS+^TM^ Premix, BD Biosciences, Franklin Lakes, NJ, USA), 50 µg/mL ascorbic acid, 100 nM dexamethasone (Sigma-Aldrich, Merck KGaA, Darmstadt, Germany), 50 ng/mL transforming growth factor (TGF)-ß1 (Peprotec, Hamburg, Germany) and 50 ng/mL insulin-like growth factor (IGF)-1 (R&D Systems, Minneapolis, MN, USA). Cells were cultivated for three days at 37 °C, 5% CO_2_, and 5% O_2_ without medium change. Afterwards, medium was replaced with DMEM containing 1% Pen/Strep, 1% Amphotericin B, 1% ITS+^TM^, 50 µg/mL ascorbic acid, and 100 nM dexamethasone. Subsequently, petri dishes containing cell-seeded scaffolds were placed in the wells of the electrical stimulation device (Figure 3). The stimulation device was placed into the incubator at 37 °C, 5% CO_2_, and 5% O_2_ and connected to the function generator via a timer. The voltage was adapted by measuring the RMS voltage at the electrodes with a voltmeter (Voltcraft plus VC-960, Hirschau, Germany). In the following, we always report the RMS value. The cell seeded scaffolds were stimulated three times each day for 45 min within eight hours over a period of seven days without media exchange. Unstimulated cells served as controls.

### 2.4. Cellular Activity

For the detection of metabolic active cells, the water-soluble tetrazolium salt (WST-1) assay (Roche GmbH, Grenzach-Wyhlen, Germany) was implemented. The tetrazolium salt WST-1 was reduced by mitochondrial dehydrogenases to formazan producing a color change from red-orange to yellow. For this assay, one scaffold of each petri dish was transferred into a 12-well plate. 500 µL of a 10% dilution of WST-1 reagent with DMEM was added to the scaffolds. After one hour of incubation at 37 °C and hypoxic conditions, 100 µL of each well was transferred into a 96-well-plate as triplicates. Absorption at a wavelength of 450 nm and at a reference wavelength of 630 nm was measured compared to a blank with the multimode plate reader Infinite 200 pro (Tecan Group Ltd., Maennedorf, Switzerland).

The scaffolds that were used for WST-1 were washed with phosphate-buffered saline (PBS, Biochrom AG, Berlin, Germany) and utilized to determine the DNA content per scaffold. Therefore, scaffolds were digested for 45 min at 37 °C in an incubation shaker (KS 4000 I control, IKA^®^-Werke GmbH & Co. KG, Staufen, Germany) in 1.5 mL reaction tubes (Sarstedt AG & Co. KG, Nuembrecht, Germany) filled up with 500 µL Collagenase A (Roche GmbH, Grenzach-Wyhlen, Germany). For quantifying DNA from these isolated cells, the peqGOLD Tissue DNA Mini Kit (VWR International GmbH, Darmstadt, Germany) was used. This kit is based on the principle of selective binding characteristics of silica membranes. After cell lysis, DNA was bound to a silica matrix in a spin column due to polar interaction. Washing steps to remove proteins, RNA and other impurities were performed according to the manufacturer’s instructions. Finally, DNA was eluted in 30 µL of elution buffer. DNA concentrations were quantified via absorption by a photometer (Tecan Group Ltd., Maennedorf, Switzerland) at a wavelength of 260 nm.

### 2.5. Gene Expression

Gene expression rates of specific genes modulating de-differentiation, chondrogenic differentiation and hypertrophy in stimulated and unstimulated cells were determined by quantifying mRNA levels. For this purpose, scaffolds were digested in 500 µL collagenase A to further isolate total RNA with the peqGOLD Total RNA Kit (VWR International GmbH, Darmstadt, Germany) according to the manufacturer’s recommendations. RNA was eluted in 30 µL of RNAse free water (Carl Roth GmbH & Co. KG, Karlsruhe, Germany) and RNA concentrations were measured via NanoQuant™ plate and TECAN Reader (both: Tecan Group Ltd.).

By reverse transcription PCR (RT-PCR), RNA samples were transcribed to cDNA. RT‑PCR was performed in a thermocycler (Biometra GmbH, Goettingen, Germany). Using the High Capacity cDNA Reverse Transcription Kit (Thermofisher Scientific), 200 ng RNA of each sample was dissolved in 10 µL water and 10 µL of the master mix were added. After RT-PCR, 20 µL of RNAse free water was added to the transcribed cDNA.

The semi-quantitative polymerase chain reaction (qPCR) is based on the principle of common PCR to amplify DNA double strands. Hereby, qPCR works with fluorescence dyes to quantify amplificated genes. For this study, the innuMIX qPCR MasterMix SyGreen (Analytik Jena, Jena, Germany) was used. The fluorescence dye Sybr Green intercalates specifically in double stranded DNA. According to instructions, 1 µL of amplificated cDNA was mixed with 9 µL of a master mix containing 2x innuMIX, forward primer (12 µM), reverse primer (12 µM) and DEPC-water. The used primer sequences are mentioned in Table 1. QPCR was done in a qTOWER 2.0 (Analytik Jena) using the following conditions: 95 °C for the initial 2 min and 39 cycles, of 5 sec at 95 °C and 25 s at 60–65 °C. A cycle of threshold (Ct) of 28 was set as the limit. Relative expression of each mRNA compared to the housekeeper β-Actin was calculated according to the equation ΔCt = Ct_target_ − Ct_β-Actin_. The relative amount of target mRNA in unstimulated cells and stimulated cells was expressed as 2^(−ΔΔCt)^, where ΔΔCt_stimulation_ = ΔCt_stimulated_ − ΔCt_unstimulated_.

### 2.6. Protein Expression

Protein expression was determined through the measurement of soluble proteins in the supernatant of stimulated and unstimulated cells.

Collagens mainly exist as collagen fibrils. To form these arrangements, collagens are released as procollagens. From these precursor molecules, the carboxy-terminal and the amino-terminal ends are cut off by procollagen peptidases for embedding the mature proteins into the fibrils. In this context, the release of the amino-terminal and carboxy-terminal ends can be used to measure the synthesis rate of collagen. For measuring the amount of type I C-terminal collagen propeptide (CICP), the MicroVue CICP ELISA (Quidel, San Diego, CA, USA) was used according to manufacturer’s instructions. A standard curve was carried along to determine protein concentrations within samples. Absorption was measured at 405 nm using a Tecan microplate reader (Tecan Group Ltd.). The Collagen Type II Synthesis ELISA (IBEX, Montréal, QC, Canada) was used to measure the concentration of type II C-terminal propeptide (CIICP). The assay procedure was performed according to the manufacturer’s instructions. Absorption was measured at 450 nm using a Tecan microplate reader (Tecan Group Ltd.).

For the detection of glycosaminoglycans (GAG), the Blyscan Sulfated Glycosaminoglycan Assay^TM^ (biocolor, Carrickfergus, UK) was used. Based on the binding of 1,9-dimethylmethylene blue to protein-free sulfated glycosaminoglycan chains, the assay creates an environment that allows specific labelling. Samples were digested overnight at 65 °C by papain (20 units/mg in 0.2 mol/L sodium phosphate buffer with pH = 6.4, Sigma Aldrich, Merck KGaA). Subsequently, the assay was implemented as recommended by the manufacturer and absorption was measured at 656 nm using a Tecan microplate reader (Tecan Group Ltd.).

All protein data were normalized to total protein content. Total protein content was measured using the Qubit^®^ Protein Assay (Thermo Fischer Scientific Inc.). Proteins are labelled via a fluorescent dye and the fluorescence level was quantified with the Qubit Fluorometer (Thermo Scientific Scientific Inc.). This device quantifies protein expression via a standard curve created by provided standards. The assay was carried out according to the manufacturer’s instructions.

### 2.7. Data Illustration and Statistics

For all experiments, a minimum of four independent donors was used. All results were plotted as boxplots using GraphPad Prism 7 (Graphpad Software Inc., San Diego, CA, USA). Median, 25%-Quartile, 75%-Quartile and whiskers from minimum to maximum were shown. All statistical tests were performed by GraphPad Prism 7. The outlier test ROUT was performed to detect outliers for boxplots and statistical analysis. Gene expression data are depicted as the percentage of 2^(−ΔΔCt)^ for better visualization of the changes with the unstimulated control set as 100%. The underlying statistical analysis was performed with the ΔΔCt-values. For the statistical analysis of protein data, the values of the specific protein amount normalized to total protein content were used. Comparison of the stimulation parameters (unstimulated, 100 mV and 1 V) within the same stimulation group (OA or non-degenerative chondrocytes) was performed with the Friedman test as these experiments were performed in parallel for the same chondrocyte donor. The paired analysis allows taking into account the inter-individual variation of the donors. For comparing the two samples of the same stimulation parameter between OA and non-degenerative chondrocytes, the Mann-Whitney-U-test was performed. The significance level was set to a level of *p* < 0.05.

## 3. Results

### 3.1. Cellular Activity

**Figure 4:**
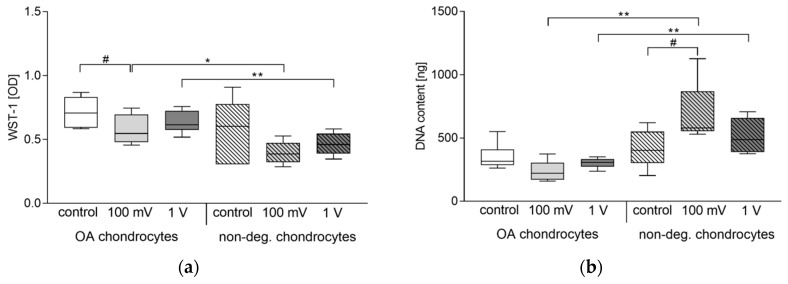
Cellular activity of human chondrocytes following electrical stimulation with 1 kHz and either 100 mV or 1 V. Chondrocytes derived from non-degenerative (*n* = 4) or osteoarthritic (OA) cartilage (*n* = 6) were seeded on collagen scaffolds and stimulated over a period of seven days. Afterwards, metabolic activity was determined via water-soluble tetrazolium salt (WST-1) assay (**a**) and DNA content was analyzed by peqGOLD Tissue DNA Mini Kit (**b**). Data are presented as boxplots. Statistical analysis within a stimulation group was performed with Friedman test (^#^ *p* < 0.05). To compare two samples between OA and non-degenerative chondrocytes, Mann-Whitney-U-test was performed (* *p* < 0.05, ** *p* < 0.01).

Compared to unstimulated cells, the metabolic activity (represented through WST-1 conversion) was reduced after electrical stimulation in OA and non-degenerative chondrocytes. For OA cells, this decrease was significant following stimulation with 100 mV (*p* = 0.0281) (Figure 4a). Comparing both cell types, a significant difference was detectable after stimulation with 100 mV (*p* = 0.0303) and 1 V (*p* = 0.0087). Here, the metabolic activity of chondrocytes derived from non-degenerative hyaline cartilage was reduced compared to OA chondrocytes.

DNA content of non-degenerative chondrocytes was significantly increased after stimulation with 100 mV (*p* = 0.0133) compared to unstimulated cells (Figure 4b). In contrast, the DNA content of chondrocytes isolated from osteoarthritic cartilage (OA chondrocytes) was slightly decreased after stimulation with 100 mV without statistical significance. Similar to metabolic activity, a significant difference between both cell types was detectable following electrical stimulation (both: *p* = 0.0043). In contrast to the metabolic activity, DNA contents of stimulated non-degenerative chondrocytes were higher compared to stimulated OA chondrocytes.

### 3.2. Induction of Chondrocytic Differentiation

**Figure 5:**
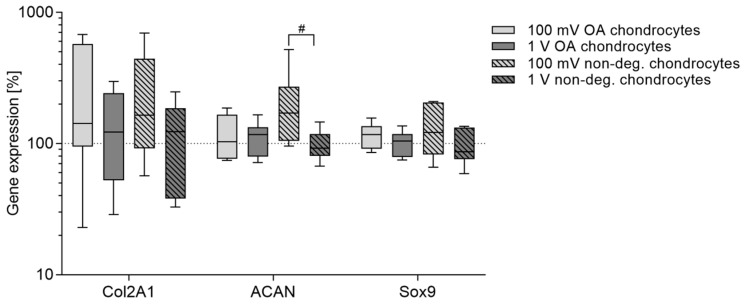
Relative gene expression of chondrogenic differentiation markers in human chondrocytes after electrical stimulation with 100 mV and 1 V at 1 kHz. Chondrocytes derived from non-degenerative (*n* = 4) or osteoarthritic (OA) cartilage (*n* = 6) were seeded on collagen scaffolds and electrical stimulation was performed over a period of seven days. Afterwards, RNA was isolated to determine the gene expression of chondrogenic differentiation markers via semi-quantitative polymerase chain reaction (qPCR). Data are presented as boxplots of the percentage of 2^(−ΔΔCt)^ related to unstimulated cells (100%). Statistical analysis within a stimulation group was performed with Friedman test by using the ΔΔCt-values (^#^ *p* < 0.05).

Regulation of chondrogenic differentiation markers following electrical stimulation was carried out for collagen II (*Col2A1*), aggrecan (*ACAN*), SRY-box (*Sox*) 9, and collagen IX (*Col9A1*). Due to the high intra-individual variability of cells, no statistically significant differences between the control groups and the electrically stimulated groups were detected. Electrical stimulation with 100 mV led to favorable results in non-degenerative chondrocytes compared to OA chondrocytes. Although *Col2A1* transcripts did not reach statistical significance, an upregulation was shown following electrical stimulation with 100 mV. Additionally, an increase in gene expression rates of *ACAN* (*p* = 0.0281 compared to 1 V) in non-degenerative cells was observed (Figure 5). Electrical stimulation did not influence the mRNA of Sox9 or rather did not induce the expression of *Col9A1*.

**Figure 6:**
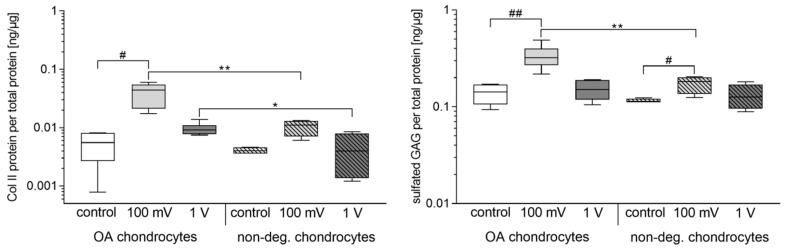
Release of collagen (Col) II and glycosaminoglycans (GAG) in human chondrocytes after electrical stimulation with 100 mV and 1 V at 1 kHz. Chondrocytes derived from non-degenerative (*n* = 4) or osteoarthritic (OA) cartilage (*n* = 6) were seeded on collagen scaffolds and stimulated over a period of 7 days. Afterwards, protein synthesis rates of Col II were determined using ELISA. Glycosaminoglycans (GAG) were analyzed by Blyscan Sulfated Glycosaminoglycan Assay^TM^. Data are presented as boxplots. Statistical analysis within a stimulation group was performed with Friedman test (^#^ *p* < 0.05, ^##^ *p* < 0.01). To compare two samples between OA and non-degenerative chondrocytes Mann-Whitney-U-test was performed (* *p* < 0.05, ** *p* < 0.01).

The release of Col II protein was significantly increased in OA chondrocytes following electrical stimulation with 100 mV (*p* = 0.0117) compared to control (Figure 6). Moreover, stimulation with 1 V led to slightly decreased protein release in OA cells compared to 100 mV. However, protein amounts were significantly enhanced compared to non-degenerative chondrocytes (100 mV: *p* = 0.0095, 1 V: *p* = 0.0381).

Compared to unstimulated controls, the release of GAG was significantly upregulated in chondrocytes when stimulated with 100 mV (OA chondrocytes: *p* = 0.0045, non-degenerative chondrocytes: *p* = 0.0400). Moreover, GAG released from OA chondrocytes was significantly higher than for non-degenerative chondrocytes (*p* = 0.0095). Following stimulation with 1 V, GAG contents were similar to those of unstimulated controls.

### 3.3. De-Differentiation and Hypertrophy after Electrical Stimulation

**Figure 7:**
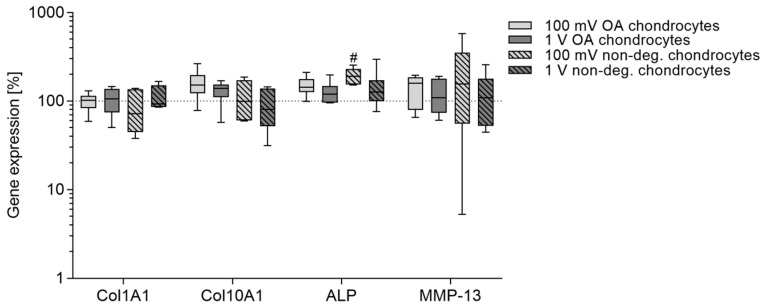
Relative gene expression rates of de-differentiation and hypertrophy markers in chondrocytes after electrical stimulation with 100 mV and 1 V at 1 kHz. Chondrocytes derived from non-degenerative (*n* = 4) or osteoarthritic (OA) cartilage (*n* = 6) were seeded on collagen scaffolds and stimulated over a period of seven days. Afterwards, the gene expression of chondrogenic de-differentiation and hypertrophy markers was determined via a semi-quantitative polymerase chain reaction (qPCR). Data are presented as boxplots of the percentage of 2^(−ΔΔCt)^ related to unstimulated cells (100%). Statistical analysis within a stimulation group was performed with Friedman test by using the ΔΔCt-values (^#^ *p* < 0.05).

To determine the influence of electrical stimulation on mRNA transcripts of important de-differentiation and hypertrophy markers, gene expression rates of collagen I *(Col1A1)*, collagen X *(Col10A1)*, alkaline phosphatase *(ALP)*, and matrix metalloproteinase *(MMP)*-13 were analyzed (Figure 7). *Col1A1* gene expression was not influenced in all stimulation groups. However, stimulation with 100 mV and 1 V led to slightly increased gene expression rates of *Col10A1* in OA chondrocytes. *ALP* mRNA transcripts were significantly upregulated in non-degenerative chondrocytes following stimulation with 100 mV compared to unstimulated control (*p* = 0.0117). Although gene expression of *MMP-13* did not reach significance in all stimulation groups, a tendency of increased expression rates could be shown after stimulation with 100 mV.

**Figure 8:**
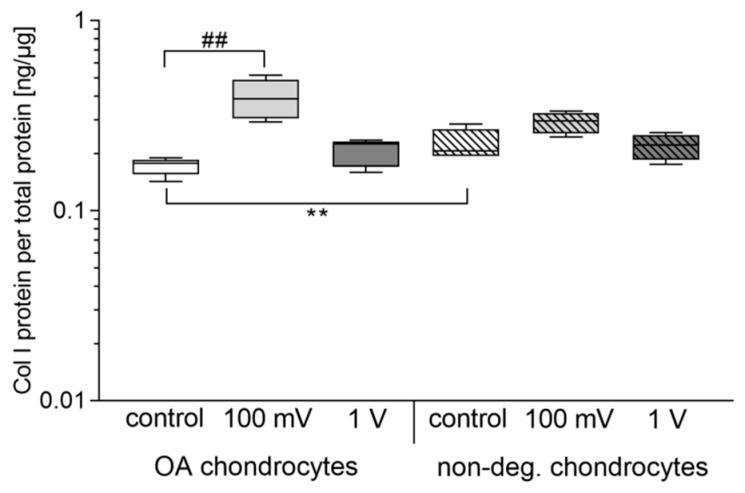
Collagen I release of human chondrocytes after electrical stimulation with 100 mV and 1 V at 1 kHz. Chondrocytes derived from non-degenerative (*n* = 4) or osteoarthritic (OA) cartilage (*n* = 6) were seeded on collagen scaffolds and stimulated over a period of seven days. Afterwards, protein biosynthesis of collagen I was detected in supernatants using the MicroVue CICP assay. Data are presented as boxplots. Statistical analysis within a stimulation group was performed with the Friedman test (^##^ *p* < 0.01). To compare two samples between OA and non-degenerative chondrocytes Mann-Whitney-U-test was performed (** *p* < 0.01).

In contrast to collagen I gene expression rates, significant changes could be determined for the release of Col I protein (Figure 8). A significant difference between unstimulated chondrocytes was observed (*p* = 0.0095). Here, non-degenerative chondrocytes released more Col I protein than OA chondrocytes. For OA chondrocytes, stimulation with 100 mV resulted in significantly enhanced protein levels (*p* = 0.0016). Additionally, an upregulated protein synthesis rate was also detectable in non-degenerative cartilage cells after stimulation with 100 mV but did not reach statistical significance. However, the release of Col I protein was slightly reduced compared to OA chondrocytes stimulated with 100 mV. Stimulation with 1 V led to similar protein levels as in unstimulated controls.

## 4. Discussion

The treatment of articular cartilage defects remains a major challenge in orthopedic surgery. The main problem in cell-based therapies like ACI or MACI is the de-differentiation of chondrocytes during ex vivo expansion [8,9,10] resulting in the formation of fibrocartilage tissue after re-implantation [11]. To counteract de-differentiation of cartilaginous cells, electrical stimulation seems to be an effective approach [16,17,18,19,20]. In 1974, Baker et al. [27] investigated the effect of electrical stimulation in a defect model of hyaline cartilage in an animal model showing an enhanced healing process with regard to cell proliferation and ECM synthesis compared to unstimulated control animals. In further in vitro analyses, Brighton et al. [28] found that metabolism of bovine chondrocytes and cartilage tissue is affected at specific field strengths of the applied electric field. In addition, this working group showed that articular chondrocytes reacted to appropriate electric fields with proliferation and upregulation of gene expression of ECM components [29]. Nevertheless, cellular reaction included the influx of Ca^2+^ into chondrocytes through voltage-gated calcium channels resulting in transduction by calmodulin, calcineurin, and nuclear factor of activated T-cells (NF-AT) [30]. All these phenomena can probably be traced back to the structure of cartilage tissue. The organization and resulting properties of hyaline cartilage are mainly determined by the main components of the ECM collagen and proteoglycans. Since hyaline cartilage is composed of different layers, these zones also contain different properties of the ECM and the matrix-forming chondrocytes [1]. This natural inhomogeneity seems to play an important role in the amplification of signal transduction mechanisms to chondrocytes. The resulting electrochemical characteristics of hyaline cartilage can be derived from the displacement of unbound cations (especially Na^+^, Ca^2+^, etc.) along the relatively fixed negative charges (e.g., SO_3_^−^ and COO^−^) present in the proteoglycans. This results in phenomena such as streaming and diffusion potentials as well as charge-dependent osmotic swelling pressures. These and further properties cause external electric signals leading to intracellular signals [21] having an influence on cell proliferation and formation of ECM components. However, it remains unclear which electric field is the most suitable for stimulating chondrocytes. A consensus in the literature revealed the use of sinusoidal alternating current with a frequency of 60 kHz [28,31,32]. Nevertheless, stimulation with a frequency of 1 kHz showed promising results [19,33]. A great discrepancy might exist in the use of electric field strength, different studies varied in their statements about the best value. The most common value for the electric field strength is 20 mV/cm postulated by Brighton et al. [31]. This is in contrast to the study of Vaca-González et al. [32] who indicated an electric field between 4 mV/cm and 8 mV/cm depending on the type of application.

The aim of our present study was to characterize the influence of capacitively coupled alternating electric fields on human chondrocytes with respect to the re-differentiation of expanded cells in vitro. For this purpose, OA and non-degenerative chondrocytes were seeded on collagen-based scaffolds and exposed to the electric field for seven days to determine cellular activity, gene expression rates of chondrogenic and hypertrophic markers as well as the release of important matrix proteins. The used stimulation parameters like sinusoidal signal form, stimulation time, frequency as well as hypoxic culture conditions and the use of collagen-based scaffolds have been selected on the basis of previous investigations in our working group [19,25,34]. In contrast to our previous works, we had now chosen a capacitive coupling of the electric fields. The great advantage of capacitively coupled systems is the prevention of electrochemical effects due to a missing direct contact of the electrodes and the electrolyte. Thus, in comparison to direct contact systems, negative effect on the cell network caused by a change in the electrochemical environment (pH, temperature, dissolved metal ions) may not arise [22,35]. However, compared to direct stimulation systems, the main disadvantage is the necessity of very high energy input to form comparable electric fields within the stimulation chamber.

Due to the avascular nature of hyaline cartilage, the hypoxic cell culture conditions create a more physiological environment [19,36]. The use of 3D structures such as scaffolds also contributes to this [26,37,38,39]. However, it should also be kept in mind that collagen type I-based scaffolds, which are commonly used to treat articular cartilage defects, do not represent the natural environment for chondrogenic cells. The use of growth factors during precultivation in our study served to initially guide the expanded chondrocytes towards differentiation [37].

Although numerical simulation revealed very low electric fields within our experimental setup, results demonstrated that the application of 100 mV_RMS_ (a field of about 5.2 × 10^−5^ mV/cm acts on the cells) had a significant impact on cellular metabolism and expression of chondrogenic markers. However, it should be noted that the used electric fields have been represented by simulation so far and have to be validated. Doing this, very sophisticated equipment is required, hence validation of the test setup will be the subject of ongoing research.

The comparison to other studies is still challenging because our electric fields differed clearly from other experimental setups [40]. Compared to the studies of Brighton et al. [20] and Vaca-González et al. [17], who also used a capacitively coupled alternating electric field, our applied electric fields were of low amplitude. Brighton and his team have conducted numerous studies in recent years to investigate the effect of capacitively coupled electric field stimulation on bovine and human cartilage explants as well as chondrocytes. Since their results showed a signal-specific increase in cell proliferation, but also a decrease in proteoglycan and collagen biosynthesis [31,41], they concluded a specific relationship between signal form and biological response. Furthermore, Vaca-González et al. [32] showed that different electric fields have different influences on cell metabolism. They pointed out that the application of 4 mV/cm led to increased proliferation, while a field of 8 mV/cm resulted in increased GAG synthesis. Although our input voltages produced very low electric fields compared to other works, we were able to demonstrate significant voltage-dependent differences in matrix synthesis. Since we have also found significant differences between non-degenerative and OA chondrocytes, the cell status might be of high relevance. Interestingly, the biological response of OA chondrocytes at 100 mV was more pronounced concerning the synthesis rate of Col II and GAG but also for Col I protein.

Chondrocytes derived from non-degenerative hyaline cartilage also reacted with enhanced levels of the mentioned ECM markers, however, the increase was clearly lower than for OA cells. This might indicate a more balanced ECM turnover compared to chondrocytes derived from degenerative-altered cartilage. This aspect might be also supported by our Col I protein results. A type I collagen synthesis for the formation of hyaline cartilage would not be desirable. However, it is important to analyze this marker as it is used as a de-differentiation marker for cartilage cells. Regarding this, it is preferable that a successful re-differentiation of the cells is accompanied by a significant decrease of Col I. Comparing the two cell types, significantly more Col I protein was released in unstimulated cells from non-degenerative cartilage. However, after electrical stimulation, these cells show no increase in protein, whereas OA cells have a significantly higher affinity for the formation and release of Col I. It is, therefore, important to adapt parameters of electrical stimulation to the cell source in order to prevent the formation of fibrous cartilage. Nevertheless, for clinical translation it must be noted that parameter optimization is essential, as cartilage defects are associated with OA progression. We have also stimulated both cell types with 100 mV_RMS_ and 1 V_RMS_ at a frequency of 60 kHz, resulting in an electric field of 0.003 mV/cm or rather 0.03 mV/cm. Here, it was shown that the effects on Col I and Col II protein biosynthesis, as well as release of GAG, were clearly reduced or no longer detectable compared to stimulation with 1 kHz (see Supplement 1). Therefore, it can be assumed that both a voltage- and frequency-dependent influence on the cells appeared to be present during electrical stimulation. Considering the electric fields which affect the cells, chondrocytes seemed to show a higher sensitization to the lower applied voltage of 100 mV_RMS_ (5.2 × 10^−5^ mV/cm) compared to the ten times higher used field at 1 V_RMS_. In contrast to the literature and our own investigations with a frequency of 60 kHz and 1 V_RMS_ (leading to an electric field of 0.03 mV/cm), this very low electric field seemed to be more effective for chondrocytic differentiation. So far, we cannot explain this phenomenon, but we assume that different mechanisms influence cell behavior. Whether the low electric fields influence either voltage-dependent channels or other structures/mechanisms that lead to downstream signaling needs to be clarified in further studies. It is possible that the stimulation in our approach leads to undesired overstimulation of the cells.

Another important aspect is the use of growth factors for chondrogenic differentiation. If cartilage implants are used [20], it can be assumed that essential growth factors are embedded in the matrix [42]. However, once the cells are removed from their 3D environment, the cells do not have access to these factors. This circumstance has to be considered in cell culture. For our experiments, we incubated the chondrocytes for 72 h with the chondrogenic growth factors IGF-1 and TGF-β1 in order to give an initial boost to intrinsic synthesis. During electrical stimulation, the cells were incubated without these factors. Due to the increased synthesis performance of the ECM components compared to the control, it can be assumed that the electric fields were sufficient to maintain the re-differentiation status. In this context, recent studies have shown that human dermal fibroblasts can also be directly reprogrammed into hyaline chondrogenic cells by electrical stimulation. Lee et al. were able to show that electrical stimulation with a frequency of 5.0 Hz and an electric field strength of 5 V/cm (applied with a commercial system) could enhance expression of chondrogenic markers, such as type II collagen, aggrecan, and Sox9 by a concomitant decrease of type I collagen without the addition of exogenous growth factors or gene transduction [43]. Considering this in further studies, it would be interesting to determine the release of specific growth factors following electrical stimulation to identify additional important pathways influenced by electric fields.

For future clinical application, further investigations are required to elucidate the signaling pathways in detail which occurred during stimulation. To increase the chondrogenic response of expanded, de-differentiated chondrocytes, combinations with other approaches could be beneficial. Here, mechanical stimulation of chondrocytes could be a promising approach since this stimulation form is well studied [17] and pro-chondrogenic effects were observed even in osteoarthritic tissue [44,45]. Due to the interaction of mechanical and electrical properties in native cartilage, a combination of electrical and mechanical stimulation might reinforce effects on each other.

## 5. Conclusions

Our results demonstrated that human chondrocytes derived from non-degenerative and OA hyaline cartilage were sensitized by low capacitively coupled electric fields (100 mV_RMS_ resulting in 5.2 × 10^−5^ mV/cm) resulting in altered cellular activity and the formation of extracellular matrix components. In addition to voltage-dependent differences, cell type-specific reactions in the synthesis rate of Col II, GAG, and Col I were observed. The underlying molecular mechanisms, which are responsible for downstream signaling at low electric fields, have to be identified in further investigations. Nevertheless, analysis of further electrical stimulation parameters should be done to induce optimal hyaline characteristics of ex vivo expanded human chondrocytes.

## Figures and Tables

**Table 1:** Overview of used primers for qPCR.

Gene		Primer Sequence	Description/Function
β-Actin (ACTB)	forward	5′-CTTCCTGGGCATGGAGTC-3′	Housekeeping gene
	reverse	5′-AGCACTGTGTTGGCGTACAG-3′	
Collagen II (Col2A1)	forward	5′-AATGGTGGCTTCCATTCAG-3′	Main macromolecule of the ECM of cartilaginous tissue
	reverse	5′-GTGATGTTCTGGGAGCCTTC-3′	
Aggrecan (ACAN)	forward	5′-ACAAGGTCTCACTGCCCAAC-3′	Proteoglycan of ECM
	reverse	5′-AATGGAACACGATGCCTTTC-3′	
SRY-box 9 (Sox9)	forward	5′-AGTACCCGCACCTGCACAAC-3′	Transcriptional factor mediating chondrocytes phenotype and cartilage homeostasis
	reverse	5′-CGCTTCTCGCTCTCGTTCAG-3′	
Collagen IX (Col9A1)	forward	5′-AACAGTGAAGGGGTCGTGAG-3′	Important function for integrity and stability of the cartilage
	reverse	5′-TGGAATTGACAGGGAATCTGGG-3′	
Collagen I (Col1A1)	forward	5′-ACGAAGACATCCCACCAATC-3′	De-differentiation marker
	reverse	5′-AGATCACGTCATCGCACAAC-3′	
Collagen X (Col10A1)	forward	5′-GAACTCCCAGCACGCAGAATC-3′	Hypertrophic marker
	reverse	5′-AGTGGGCCTTTTATGCCTGT-3′	
Alkaline phosphatase (ALP)	forward	5′-CATTGTGACCACCACGAGAG-3′	Transcriptional factor mediating hypertrophy
	reverse	5′-CCATGATCACGTCAATGTCC-3′	
Matrix metallopeptidase (MMP)-13	forward	5′-CACGCATAGTCATATAGATACT-3′	Degradation enzyme for Collagen II
	reverse	5′-CTGGAGATATGATGATACTAAC-3′	

## Supplementary Materials

The following are available online at https://www.mdpi.com/2077-0383/8/11/1771/s1, Figure S1: Release of collagen (Col) I (de-differentiation marker), Col II and glycosaminoglycans (GAG) (both differentiation marker) from human chondrocytes after electrical stimulation of 100 mV and 1 V at 60 kHz.
