# Supplementary material for: Re-Differentiation Capacity of Human Chondrocytes in Vitro Following Electrical Stimulation with Capacitively Coupled Fields
Source: J Clin Med. 2019 Oct 24;8(11):1771. doi: 10.3390/jcm8111771 (PMC6912508; doi:10.3390/jcm8111771)
Supplement: Supplementary file 1 [file jcm-08-01771-s001.pdf]

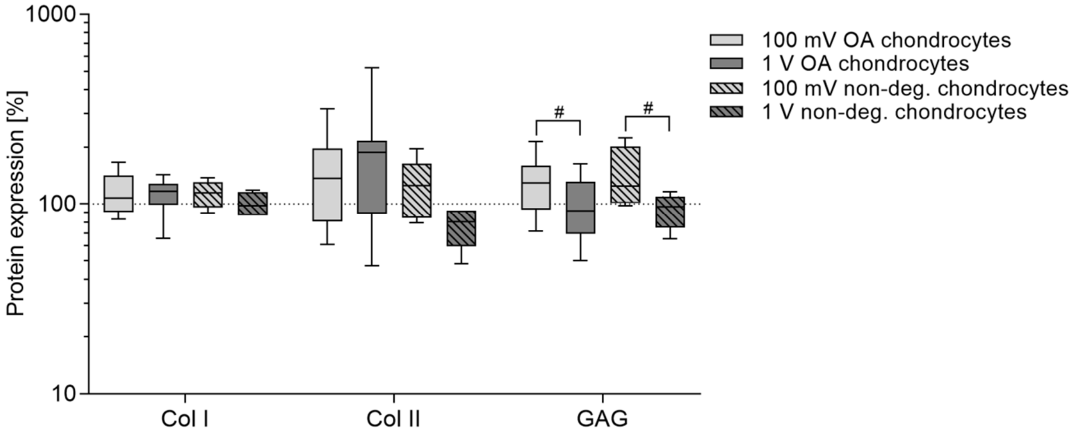

Supplement Figure S1. Release of collagen (Col) I (dedifferentiation marker), Col II and glycosaminoglycans (GAG) (both differentiation marker) from human chondrocytes after electrical stimulation 100 mV and 1 V at 60 kHz. Chondrocytes derived from non-degenerative (*n* = 6; 3 male and 3 female donors, 43 ± 10 years) or osteoarthritic (OA) cartilage (*n* = 6; 2 male and 4 female donors, 68 ± 9 years) were seeded on collagen scaffolds and stimulated over a period of seven days. Afterwards, biosynthesis of the ECM components Col I, Col II and GAG was detected in supernatants using specific assays. Data are presented as boxplots (related to unstimulated cells). Statistical analysis within stimulation group was performed with Friedman test ($^{\#}p < 0.05$).
